# “They seemed to forget about us little people”: the lived experiences of personal care attendants during the COVID-19 pandemic

**DOI:** 10.3389/fsoc.2025.1460307

**Published:** 2025-02-14

**Authors:** Carrie Wendel, Darcy L. Sullivan, Jennifer Babitzke, Tracey A. La Pierre

**Affiliations:** ^1^School of Social Welfare, University of Kansas, Lawrence, KS, United States; ^2^Department of Population Health, University of Kansas Medical Center, Kansas City, KS, United States; ^3^Department of Sociology, University of Kansas, Lawrence, KS, United States

**Keywords:** COVID-19, personal care attendants, long term services and supports, home and community based services, mixed methods, Medicaid

## Abstract

**Background:**

Personal care attendants (PCAs) provided essential care and support to home care clients during the COVID-19 pandemic and thus were a vital part of the pandemic response in helping to keep older adults and individuals with disabilities out of nursing homes. Furthermore, they are one of the largest and fastest growing workforces in the United States. Yet this essential workforce received little attention during the pandemic. Guided by feminist theories on caregiving and the principles of community-based participatory research, this study examined the experiences of PCAs during the COVID-19 pandemic.

**Methods:**

Data from 78 in-depth interview participants representing Medicaid-Funded Home and Community-Based Services (HCBS) PCAs, clients, family caregivers, and service providers in Kansas, United States, as well as additional data from 176 PCA survey participants were analyzed. Findings from this interactive, convergent, mixed-methods study were integrated by theme using the weaving approach.

**Results:**

Four major themes emerged from the analysis: (1) PCAs remained in this field during the pandemic out of a commitment to their clients; (2) PCAs were undervalued and invisible as an essential workforce; (3) direct care work had an emotional toll on PCAs during the pandemic; and (4) PCAs have mixed feelings about their satisfaction with the job, and, as good workers quit, they were difficult to replace.

**Discussion:**

PCAs held professional-level responsibilities without the recognition or pay of a professional. The pandemic had mixed impacts on job stress and satisfaction, suggesting that the intrinsic rewards of the job and social support had a protective impact. However, intrinsic rewards are not enough to retain this workforce, and the growing PCA workforce shortage leaves many clients having to choose between no care and poor care. Our findings indicate that institutions and systems must better support and recognize this essential workforce to build and maintain a quality in-home care services system.

## Introduction

Personal care attendants (PCAs) provide essential hands-on support to older adults and people with disabilities in their homes by helping with tasks such as cooking, bathing, housekeeping, and shopping. In addition to providing support for activities of daily living, PCAs are also a source of emotional support for the clients they serve (Franzosa et al., 2019). These vital supports allow people with disabilities to continue living in the community and maintain their independence (Spillman, 2016), which was of critical importance during the COVID-19 pandemic as institutions became significant focal points for transmitting the virus. PCAs are now the largest workforce in the United States and one of the fastest growing occupations (PHI, 2023; Van Dam, 2024); however, they are also relatively invisible as an unlicensed workforce that works behind closed doors in private home settings. The purpose of this article is to explore PCA experiences in providing essential care during the pandemic, utilizing mixed-method interview and survey data from a broader study on the Medicaid Home and Community-Based Services (HCBS) system response to the COVID-19 pandemic in Kansas, United States.

## Context and background

### Key stakeholders and terminology in the HCBS system

Our study focuses on PCAs who provided Medicaid-funded HCBS personal care services during the pandemic in Kansas. Other stakeholders include clients, family caregivers, and providers. HCBS is the program that provides long-term services and supports (LTSS) in home and community settings as an alternative to institutional care. This is a complex system involving many stakeholders and industry-specific terminology; therefore, we detail the key players and terms here.

Adult HCBS waiver programs in Kansas include the Frail Elderly, Physically Disabled, Brain Injury, and Intellectual and Developmental Disability programs. We refer to those who receive these HCBS services as clients. In Kansas, HCBS clients can choose agency-based or self-directed care for their personal attendant care. In the traditional agency-based model, clients sign up with a home care agency who hires and manages the PCAs going into client homes. The home care agency is the PCA’s employer in this model. In the self-directed model, clients can hire, fire, and manage their own workers. They sign up with a financial management service provider to manage PCA payroll. Financial management service providers also provide information and assistance to HCBS clients in carrying out their employer role but are not the employer. We also included other community-based providers who help support the delivery of HCBS by providing services such assisting clients with service applications and referrals or supporting providers. We use the term provider to refer to home care agency, financial management services, and other community providers at the organizational level and distinguish provider type when relevant.

There is little consistency in job titles for the direct support workforce that provides hands-on LTSS care. Direct support workers who specifically work in home care settings may be referred to as caregivers, home health aides, home care aides, direct support professionals, or personal care aides/attendants. Among these titles, only home health aides and personal care aides have a U.S. Bureau of Labor Statistics Occupational Code. We have chosen to use the personal care attendant (PCA) title because it more clearly delineates a non-certified worker who provides care in private homes, although the participants in our study used all of these different job titles. HCBS clients in the self-directed program can hire friends and family as their PCAs, so when relevant, we demarcate paid family caregivers as related PCAs. Unpaid family caregivers also provide valuable support to HCBS clients, sometimes managing and overseeing their paid care, and were included in our study. We refer to them as family caregivers. Pay is often the only thing that differentiates the roles and responsibilities of a paid vs. unpaid family caregiver, but since paid family caregivers are officially workers within the HCBS system, we call them PCAs.

Medicaid is privatized in Kansas, in which three for-profit managed care organizations administer the program. Managed care organization care coordinators develop care plans and authorize services for HCBS clients, including setting the approved PCA tasks and hours. Managed care organization representatives, including care coordinators, were not included as subjects in our study; however, they were frequently referenced by our study participants.

### Medicaid HCBS and COVID-19 policy in the United States and Kansas

It is important to understand the policy context that shapes the wages, benefits, and job conditions for the PCA workforce. The United States is known for high rates of inequality, limited labor protections, and meager social benefits compared to other advanced industrial nations. Medicaid, the means-tested insurance program for low-income individuals, is the primary payer of LTSS in the U.S (Chidambaram, 2022). Medicaid is funded and administered through a state–public partnership, which results in wide variation across states in how these programs are operated. In Kansas, the state legislature sets the reimbursement rates that drive wages for the direct support workforce.

Turning to benefits, the Affordable Care Act was intended to provide universal healthcare coverage to Americans through a combination of private and public healthcare coverage. A key component of the Affordable Care Act was that Medicaid would be expanded to include more low-income Americans, and then, government subsidies were allotted to help cover the cost of private health insurance through the Affordable Care Act Marketplace for those with moderate-level incomes who do not qualify for Medicaid. However, the U.S. Supreme Court ruled that states could not be required to expand Medicaid (MACPAC, 2022). This resulted in a healthcare coverage gap for non-expansion states for those who earn too much to qualify for Medicaid but not enough to qualify for Affordable Care Act Marketplace subsidies. Kansas is one of 10 states that has still not expanded Medicaid (KFF, 2024). As a low wage workforce, PCAs often fall in this coverage gap (PHI, 2023). Of additional importance for understanding the conditions of this occupation, there is no guaranteed paid leave in the United States, with only 39% of the lowest-wage workers having access to paid sick leave (Gould and Wething, 2023).

The United States federal government invested more than $5 trillion into the COVID-19 pandemic response (Parlapiano et al., 2022). The initial federal financial aid package was the Coronavirus Aid, Relief, and Economic Security Act of 2020, designed to support worker safety and stabilize the economy. Nursing homes received direct Coronavirus Aid, Relief, and Economic Security Act funding to support their workforce, but HCBS providers had to apply for these funds (Wendel et al., 2023). The next federal financial aid package was the American Rescue Plan Act of 2021, which included funds allocated to states for their HCBS programs. States had a lot of latitude in how to use these funds within federal guidelines and oversight. Kansas received an estimated $102 million in American Rescue Plan Act funding, of which over $50 million was distributed to the HCBS PCAs in the form of bonuses (Heydon, 2023). This was designed as a one-time bonus since prolonged wage increases would require a long-term commitment from the state legislature.

### Background theory and literature

Feminist theories on caregiving have highlighted the essential nature of care work and reproductive labor for the health, wellbeing, and survival of human society, while exploring the myriads of ways this essential labor has been devalued and exploited (e.g., Tronto and Fischer, 1990; Glenn, 2010; Tronto, 2013; Fraser, 2016; Folbre, 2024). Historically, Western society has deemed domestic labor in the private sphere as inferior to the paid labor performed within the male-dominated, public marketplace (Engels, 1884; Glenn, 1992; Tronto, 2013; Tong and Botts, 2017). Caregiving, whether paid or unpaid, is devalued and made invisible because it takes place within the household (Engels, 1884; Glenn, 1992; Tronto, 2013; Tong and Botts, 2017; Bandini et al., 2021). Traditional market-based economic measures fail to capture the true value of care work, which is undervalued as unpaid or underpaid labor. The economy relies on care work being free or cheap to sustain the workforce while minimizing labor costs, enabling greater profitability and economic productivity by exploiting the essential role of caregiving (Tronto, 2013; Fraser, 2016; Folbre, 2024).

Care work is also devalued by false assumptions that it is unskilled labor that comes naturally to women, but as Tronto and Fischer, (1990) illustrate, naturalistic assumptions about caring overlook that fact that quality care requires time, material resources, knowledge, and skill to carry out. Non-family caregiving performed by domestic laborers also has deep roots in slavery and indentured servitude (Glenn, 1992) magnifying the forced and exploitative nature of care work. The consequences of this history are evident in the demographics of both paid and unpaid caregiving for those with LTSS needs today. Women, particularly women of color and immigrant women, are overrepresented in unpaid and paid care-related work globally, including the United States’ PCA workforce which is 87% female (PHI, 2021). This reflects the “dual devaluation of caring,” discussed by Glenn (2010), in which society assigns caregiving to our most disadvantaged and powerless citizens because we do not value care, and, in turn, care work is reinforced as unskilled resulting in a cycle in which caring and caregivers are simultaneously devalued.

This devaluing of care is evidenced by the low wages and lack of benefits for formal caregivers (Blum and Mathis, 2021; Scales, 2021). In 2021, the median annual income for PCAs providing in-home care in the United States was approximately $18,100 (PHI, 2021). The median annual income for PCAs in Kansas is notably lower than the national average at $16,131 (PHI, 2023). For many people, this means they fall below the federal poverty level (FPL). For example, in the United States, 25% of PCAs have incomes less than 138% of the FPL and 28% of PCAs in Kansas have incomes below 138% of the FPL (PHI, 2023).

The undervaluing of care in our economic and social structures leads to secondary dependency in which caregivers become economically dependent on primary breadwinners or the state (Kittay, 1999; Glenn, 2010; Tronto, 2013). This is true of the many PCAs who struggle to support themselves financially; 53% of direct support workers rely on public assistance, such as food assistance, to afford necessities (PHI, 2021). Forty-six percent of PCAs in Kansas receive some form of public assistance (PHI, 2023). In the US, an average of 37% of the PCA workforce receives insurance through their employers, and 43% report Medicaid or Medicare as their primary form of health insurance coverage (PHI, 2021). Approximately 17% of the national PCA workforce was uninsured (PHI, 2021). In Kansas, 33% of PCAs rely on Medicaid or Medicare for health insurance coverage, while 25% are uninsured (PHI, 2023). Due to low wages, it is common for PCAs to have additional jobs. An estimated 6.41% of personal care aides hold second jobs; these estimates are approximately 35% higher than workers in other occupations (Baughman et al., 2022).

Reflecting the complex nature of care work, work-related stress is common among PCAs (Gray-Stanley and Muramatsu, 2011; Bandini et al., 2021; Maffett et al., 2022; Janssen and Abbott, 2023). Heavy workloads, abusive behaviors from clients and clients’ families (Maffett et al., 2022), and lack of job autonomy contribute to stress and exhaustion that can lead to burnout (Gray-Stanley and Muramatsu, 2011; Bandini et al., 2021). Existing research indicates that work stress is positively associated with burnout (Gray-Stanley and Muramatsu, 2011). Access to resources, such as social support, has been shown to moderate the relationship between work-related stress and burnout; similarly, locus of control or being involved in care-related decision-making also moderates the relationship between work-related stress and PCA burnout and decreases overall job dissatisfaction (Gray-Stanley and Muramatsu, 2011; Kusmaul et al., 2020). PCAs have also reported that increased wages, benefits, and training opportunities would lower work-related stress and burnout (Janssen and Abbott, 2023; Karmacharya et al., 2023).

The COVID-19 pandemic exacerbated many of the ongoing challenges experienced by the PCA workforce as demand for this underpaid and undervalued workforce grew (Blum and Mathis, 2021; Scales, 2021; Kreider and Werner, 2023). During the COVID-19 pandemic, PCAs served people with disabilities and older adults who were at-risk populations while oftentimes being at-risk of serious illness themselves (Almeida et al., 2020; Sama et al., 2021). Since PCAs were not consistently classified as “essential workers,” many PCAs reported not being able to access necessary personal protective equipment, COVID-19 testing, and vaccines (Bandini et al., 2021; Sama et al., 2021; Tyler et al., 2021; Wendel et al., 2023). As a result of these working conditions, some PCAs left the workforce, which exacerbated existing worker shortages (Blum and Mathis, 2021; Frogner and Dill, 2022), while others kept working throughout the pandemic due to financial constraints and a sense of duty to their clients (Blum and Mathis, 2021). PCAs are now one of the largest and fastest growing workforces in the United States (Van Dam, 2024) but still falling far short of meeting the growing demand for home care services (Scales, 2021). Ultimately, feminist theorists attribute the caregiver crisis to the exploitation of caregivers and failure to value care in our capitalist society (e.g., Tronto, 2013; Fraser, 2016).

## Methods

Data for this study are drawn from 78 in-depth interview participants across stakeholder groups (related PCAs *n* = 12; non-related PCAs *n* = 14; unpaid family caregivers *n* = 5; clients *n* = 27; providers *n* = 21) and survey data from 176 PCAs. These data come from a larger mixed-methods study that used in-depth interviews and surveys with HCBS clients, PCAs, family caregivers, and service providers to explore how the HCBS system in Kansas responded to the challenges of the COVID-19 pandemic. An interactive, convergent mixed-methods design was adopted. This process involved concurrent qualitative and quantitative data collection and analysis with independent interview and survey samples that iteratively informed subsequent data collection (Fetters et al., 2013). Data collection occurred between May 2021 and June 2023.

Guided by feminist theories on caregiving and the principles of community-based participatory research, all data collection tools (surveys and semi-structured interview guides) were developed with the input of a Stakeholder Advisory Board (SAB). The SAB included PCA, caregiver, client, provider, and advocate representatives and advised on all aspects of the project from research questions and data collection tools to policy implications and dissemination. This ensured research questions, methodologies, and interpretations were responsive to community needs and perspectives. Several members of the research team had relevant lived experience, either as family caregivers or prior work experience as PCAs, providing a deeply nuanced understanding of the challenges and complexities of caregiving and sensitizing them to potential research challenges such as confidentiality and privacy concerns, power dynamics, and participant vulnerabilities. The PI also drew on her prior applied research and advocacy in the HCBS system in developing the study. The diversity of experiences, perspectives, and training of the research team and the SAB provided collaborative reflexivity throughout the research process by challenging individual assumptions and revealing potential blind spots in research design and interpretation (Olmos-Vega et al., 2023). Regular research team and SAB meetings provided structured opportunities to discuss potential ethical challenges, engage in collective interpersonal, methodological, and contextual reflexivity, and were a mechanism for continuous feedback and collaborative knowledge production.

We drew on existing community connections to recruit SAB members and gatekeepers to recruit participants but also identified new partners to help fill key gaps in our community engaged design. The SAB was first convened during the proposal development stage and shared their experience from the field to help refine research questions and methodology. Initial drafts of interview guides and surveys were crafted based on the literature and expertise of the research team and then shared with SAB for further refinement. The semi-structured interview guides were adapted over time in response to initial results as well as reports from the field brought to the table by SAB members on service delivery or pandemic developments that warranted further investigation. They also advised on areas where research data could help guide policy and practice. Interviews were launched prior to the surveys, and therefore, early results also informed final revisions to the survey tools. SAB members with cognitive impairments conducted plain language review and editing of survey questions, as well as the informed consent statement. Survey questions were also tested using cognitive interviews (Beatty and Willis, 2007), in which participants were asked to verbalize their thought processes while completing the survey and respond to probing questions by a member of the research team to ensure survey questions were understood and accurately captured the intended information. Preliminary and emergent findings were discussed with the SAB providing member checking and additional contextual reflexivity (Olmos-Vega et al., 2023).

All recruitment materials were prepared in both English and Spanish, with translators available to assist Spanish speakers with interviews or surveys. Community partners were instrumental in facilitating recruitment of interview participants, along with snowball sampling and social media. Interview and survey participants were recruited independently, and selection into one sample had no influence on selection into the other. Five interview participants had previous professional encounters with a member of the research team (four providers and one PCA), and one PCA participant who had a closer professional relationship to one member of the team was interviewed by a different team member. Interviews were conducted via zoom or by phone, lasted approximately 90 min on average, and were recorded and transcribed verbatim and then deidentified. Turning to surveys, except for six PCA survey respondents recruited through social media, recruitment of PCAs for the quantitative sample was through home care agencies and financial management service providers who were willing to distribute recruitment materials to all PCAs in their organization and act as gatekeepers for recruitment in specific geographic areas. Survey data were collected using Qualtrics and analyzed using StataMP 17.

All study procedures were approved by the University of Kansas Human Subjects Protection Program (Study #:00146397), and additional care was taken to safeguard the wellbeing of research subjects. Study participants were compensated for their time. Interviews and surveys were confidential and conducted with informed consent; survey participants indicated agreement to an informed consent statement via a checkbox rather than a signature to allow surveys to be completed anonymously, and interview participants provided informed consent verbally prior to starting the audio-recording. Some interview participants requested confirmation that their interview was confidential before sharing critical or sensitive information, indicating heightened distrust of the system. Therefore, the research team took special care to de-identify the data. In addition to removing names and locations, other contextual details that could potentially identify the person to someone known to them were removed. Participants were free to withdraw from the study at any time and did not have to respond to any questions they did not feel comfortable answering. Finally, interviewers provided research participants with informational resources as appropriate, for example, where to find free personal protective equipment or COVID vaccines or shared HCBS policy or contact information to those with service-related concerns they wanted to address. The research team also had a protocol in place for responding to any abuse or serious mental health concerns that may be revealed during interviews, although this circumstance did not occur. Many participants expressed appreciation for the study and being able to voice their concerns candidly and confidentially.

Interview and open-ended survey data were analyzed by the authors using iterative, consensus-based inductive coding (Cascio et al., 2019). First-level coding was completed separately by four members of the research team. Authors discussed initial codes and any discrepancies were resolved through discussions of the data. New codes and emerging themes were regularly discussed at team meetings. Approximately one in three interviews were double-coded to strengthen intercoder consistency. Dedoose software was used for all coding.

All codes and variables related to the nature of the PCA job—what they were doing, what they were experiencing, and how they and others felt about their job—were analyzed to explore the experiences of HCBS PCAs in Kansas during the COVID-19 pandemic. These qualitative codes were analyzed across all subgroups, including PCAs, clients, providers, and family caregivers. Thus, the qualitative data captured PCA’s own experiences as well as the experiences of those who receive care from PCAs or supervise their work.

Quantitative data come from the survey sample of 176 PCAs. The PCA surveys included questions about PCA job satisfaction, stress, feelings of support and respect, intent to quit, COVID exposure and vulnerability, access to benefits, and self-reported health (see Tables 1, 2 in results for more detail on survey measures). Survey data were collected using Qualtrics and analyzed descriptively using StataMP 17.

**Table 1:** Demographic characteristics of client, PCA, and caregiver interview participants.

	Clients^*^	Non-related PCAs	Related PCAs	Family caregivers (unpaid)^*^	Total
	*n* = 27	*n* = 14	*n* = 12	*N* = 5	57
Age					
Range	23–81	21–72	45–73	55–69	21–81
Mean	54	49	59	58	54
Gender					
Male	7	2	2	0	11
Female	20	12	10	5	46
Race/Ethnicity					
Hispanic/Latino	3	1	1	1	5
American Indian or Alaska Native	2	0	0	0	2
Black	5	2	1	0	8
Asian	0	1	0	0	1
White Non-Hispanic	18	10	10	4	42

* One respondent was both a consumer and a family caregiver (middle-aged, Hispanic, white female) and is included in both categories but not double-counted in totals.

^^^One respondent was multiracial, and so, race/ethnicity subtotals are greater than total sample.

**Table 2:** Program characteristics of interview participants.

	Clients^*^	Non-related PCAs^^^	Related PCAs^^^	Family caregivers (unpaid)^*^^	Providers^^^	Other providers^^^^#^
	*n* = 27	*n* = 14	*n* = 12	*n* = 5	*n* = 13	*n* = 8
Waiver						
Brain Injury	5	1	2	0	9	5
Frail Elderly	5	9	2	1	10	5
Intellectual/Developmental Disability	5	5	11	3	9	4
Physical Disability	12	7	6	3	10	5
Care Model						
Agency-based care	6	6	1	0	8	n/a
Self-directed care	15	7	6	2	3	n/a
Both	6	1	5	3	2	n/a
Geographic Region						
Metropolitan	20	9	9	2	2	4
Non-Metropolitan	7	5	3	3	3	1
Mix/both	n/a	n/a	n/a	n/a	8	3

* One respondent was both a self-directed Physical Disability Waiver client and an unpaid family caregiver to a self-directed Physical Disability Waiver client and is included in both categories but not double-counted in the total n.

^ Some PCAs, family caregivers, and providers support multiple service recipient types, and therefore, subtotals are greater than the sample size.

# Other providers included two provider associations, four Aging and Disability Resource Centers, and two Community Developmental Disability Organizations.

Qualitative and quantitative data were integrated in our analysis using a constant, comparative method to systemically examine areas of agreement, contradiction, or expansion between datasets (Creswell and Plano Clark, 2018). Research team members worked across both the qualitative and quantitative datasets to immerse themselves in the data and identify patterns and discrepancies. The research team also met regularly to discuss emergent findings and areas in which qualitative data indicated a need to consult the quantitative data and vice versa. Qualitative and quantitative findings were integrated on a theme-by-theme basis in the text using a weaving approach (Fetters et al., 2013).

Demographic and program characteristics for the in-depth interview sample can be found in Tables 1, 2 and for the PCAs in the survey sample in Table 3. The sample is predominantly white, consistent with the racial makeup of Kansas (United States Census Bureau, 2023). This also reflects our concerted effort to recruit rural participants as the SAB advised that geography was an important dimension shaping service delivery and the pandemic response. The sample represents diverse program characteristics such as waiver type and self-directed vs. agency-based, which are also important dimensions influencing service delivery.

**Table 3:** Demographic characteristics of PCA survey respondents.

	*N* = 176%, (*n*)
Age	
Range	19–87
Mean (SD)	47.3 (1.09)
Gender	
Male	17.0 (30)
Female	82.4 (145)
Transgender/non-binary	0.6 (1)
Race/Ethnicity	
White	82.4 (145)
Black	13.1 (23)
American Indian or Alaska Native	4.0 (7)
Asian	2.8 (5)
Hispanic/Latino	2.8 (5)
Education	
Less than high school	5.7 (10)
High school graduate	24.4 (43)
Vocational, technical, or trade school	11.4 (20)
Some college, but no degree	25.6 (45)
Associate’s degree	10.8 (19)
Bachelor’s degree	10.2 (18)
Master’s, professional, or doctoral degree	8.5 (15)
Care Model	
Agency-based care	14.2 (25)
Self-directed care	76.1 (134)
Both	6.3 (11)
Missing/Ambiguous	3.4 (6)
Employment Status	
Part-time	61.9 (109)
Full-time	37.5 (66)
Length of time as a PCA	
Less than a year	15.3 (27)
1–2 years	19.9 (35)
3–5 years	23.9 (42)
6–10 years	13.6 (24)
More than 10 years	27.3 (48)
Related to a client	
No	52.3 (92)
Yes	47.7 (84)
Employed as PCA prior to the COVID-19 Pandemic	
No	20.7 (54)
Yes	63.1 (111)
Not sure	2.8 (5)
Health insurance	
Uninsured	19.9 (35)
Insurance through another employer	11.9 (21)
Insurance through spouse, partner, or parent	19.3 (34)
Marketplace	10.8 (19)
Private pay insurance or not specified	1.1 (2)
Medicaid, Medicare, Military VA	29.0 (51)

Race/ethnicity category totals more than 100% as participants could select multiple races/ethnicities. Categories of each variable may not add to 176 due to missing data.

## Results

Our findings are centered around four major themes: 1) PCAs remained in this field during the pandemic out of a commitment to their clients; 2) PCAs were undervalued and invisible as essential workers; 3) direct care work had an emotional toll on PCAs during the pandemic; and 4) PCAs have mixed feelings about their satisfaction with the job, and, as good workers quit, they were difficult to replace. Table 4 provides an overview of qualitative themes, subthemes, and exemplar quotes. Tables 5, 6 show descriptive statistics from the PCA survey related to job conditions, job satisfaction, and wellbeing.

**Table 4:** Qualitative themes, subthemes, and illustrative quotes.

Theme 1: PCAs remained in this field during the pandemic out of a commitment to their clients.	
Subthemes	Illustrative quotes
PCAs were committed to client wellbeing, rooted in the duty to care and relational bonds.	We choose to do this job because our hearts are in it not because we make money or get benefits. I could definitely get a higher paying job I just do not have the heart to leave my client not knowing the care he will receive.
PCAs were motivated to keep their clients out of nursing homes during the pandemic.	Because my client needs someone there and without us PCAs he would be in a nursing home being neglected and not cared for as needed.
PCAs went above and beyond to deliver quality, safe care during the pandemic.	Sometimes I’d stay over my time. I just clocked out when I needed [to], but if the job wasn’t done for the amount of hours they gave me, I went ahead and did it on my own free time because that’s just the way I am.
Theme 2: PCAs were undervalued and invisible as an essential workforce.	
Subthemes	Illustrative quotes
Compared to other essential workers, PCAs felt unrecognized and invisible.	When they were giving all these blessings and compliments to the nurses that are at risk in the hospital, they seemed to forget about us little people and the elderly there in the homes that are doing just as good as job as they were.
The invisibility of PCAs as essential workers impacted their access to key pandemic resources, such as access to personal protective equipment, vaccines, or hazard pay.	It felt that as an agency that provides non-medical services… we were left out. Oftentimes we would find out after the fact about programs that would hand out donated gloves and other [personal protective equipment] items. Additionally, convincing the Health Dept. that our staff needed vaccinations was a problem!
The devaluing of PCAs is evident in their low wages and poor benefits.	Having contracted COVID-19 on my [PCA] job, I had to self-isolate for 10 days, during which I could not work either of my jobs while I recovered…. Not only were we at higher risk for exposure, when we got sick, we had no sick pay or unemployment benefits and shouldered the financial consequences on our own.
The knowledge of PCAs as experts on their clients’ care needs was not well recognized or supported.	All I know is what their needs are…. These people (care coordinators) wasn’t listening to the clients’ needs… When we were out here being their eye and the voice, they would not listen to us.
Theme 3: Direct care work had an emotional toll on PCAs during the pandemic.	
Subthemes	Illustrative quotes
PCA’s work is physically, mentally, and emotionally challenging.	It’s hard work… I do not think people understand how hard it can be because we go in there every day and take care of people that have different personalities. They have different medical needs, and we have to make sure that they have everything that they need, and that we have everything we need. I’ve been pinched. I’ve been hit. I’ve had my hair pulled. I’ve been kicked.
PCAs feared contracting the virus or spreading it to their vulnerable clients.	Personally, for me, if I knew I had COVID and I gave it to someone else… and they died, I would, literally, probably never forgive myself. I would feel so bad… I was really nervous to go back to work in general.
PCAs also had to help manage the emotions, loneliness, or increased behaviors of their clients experienced in response to the pandemic	Telling the individuals they could not see their family, while driving home to my family every night. It changed me. Being the bad guy to people who did not understand. I saw behaviors in individuals that were extremely out of character because they missed their families.
PCAs experienced role overload as responsibilities increased, contributing to burnout.	[I was] tired but I did [it]…I’m in between a rock and a hard spot. This is my passion. I also feel like if I’m not there, [then] nobody else will be there…there’s just me… I had some of [my clients say], “Please do not go. Could you spend a little bit more time with me? I’m tired of being here alone.”
PCAs practiced self-care and valued emotional support, but support available was inconsistent.	-At one point, during lockdown, they sent us little packages with paperclips, a journal with little positive thoughts in it. Just little simple things. A card that said you were appreciated. [My company] is very good about letting you know we appreciate you. We’re blessed with a very special CEO. They do so much. -What support?
Theme 4: PCAs have mixed feelings about their satisfaction with the job and as good workers quit, they were difficult to replace.	
Subthemes	Illustrative quotes
PCAs qualified their sense of job satisfaction as enjoying the job but not the pay and benefits	[I am satisfied] with the job, but not the pay.
Poor wages and benefits contributed to high-quality workers leaving the field	She [former PCA] had a really good personality. She was jolly to be around. She did a fabulous job. She would come if I needed something after she had gotten home. She would come back over, just a very good person…. She left (during the pandemic) for full-time employment with benefits.
Workforce shortages combined with low pay drew poor workers to the field	I’ve had the pleasure of having—I’ve had my car stolen, I’ve had money stolen, I’ve had my meds stolen…. If you had better pay, you would get better quality of people, I hope.

**Table 5:** Job conditions and job satisfaction among PCAs.

	All (*N* = 176) %, (*n*)
“I feel respected by clients as part of their home care team.”	
Strongly agree	72.2 (127)
Somewhat agree	15.9 (28)
Neither agree nor disagree	6.8 (12)
Somewhat disagree	2.8 (5)
Strongly disagree	2.3 (4)
How has the respect you feel from clients as part of their home care team changed since before the pandemic?	
I feel more respected by my clients as part of their care team	15.9 (28)
I feel less respected by my clients as part of their care team	3.4 (6)
I do not feel there is any difference in how much I am respected by my clients as part of their care team	52.8 (93)
“Overall, my employer provided good support and assistance during the pandemic.”	
Strongly agree	25.0 (44)
Somewhat agree	6.8 (12)
Neither agree nor disagree	12.5 (22)
Somewhat disagree	1.7 (3)
Strongly disagree	4.0 (7)
How satisfied are you with your occupation as PCA?	
Extremely satisfied	60.2 (106)
Somewhat satisfied	33.0 (58)
Somewhat dissatisfied	5.1 (9)
Extremely dissatisfied	0.6 (1)
How has your satisfaction with your job changed compared to before the pandemic?	
I am more satisfied with my job	12.5 (22)
I am less satisfied with my job	5.7 (10)
There has been no change in my job satisfaction	52.3 (92)
How likely is it that you will stop working as a PCA in the next year?	
Very likely	7.4 (13)
Somewhat likely	17.0 (30)
Not at all likely	65.3 (115)
How often do you find your job as PCA stressful?	
Always	4.5 (8)
Often	18.2 (32)
Sometimes	42.6 (75)
Rarely	23.9 (42)
Never	9.1 (16)
How has your job stress changed compared to before the pandemic?	
I have less work stress now than before the pandemic	8.0 (14)
I have more work stress now than before the pandemic	10.2 (18)
I have about the same amount work stress now as before the pandemic	52.8 (93)
Did you incur out of pocket expenses to make home care services safer?	
No	65.3 (115)
Yes	33.0 (58)
Receives no benefits from employer (health insurance, paid leave, holiday pay, etc.)	
No	25.0 (44)
Yes	73.3 (129)
Were you ever exposed to COVID-19 through your job as a PCA?	
No	50.0 (88)
Yes	39.2 (69)
Not sure	10.8 (19)
Did you provide home care services to anyone while they had COVID-19?	
No	63.1 (111)
Yes	29.5 (52)
Not sure	7.4 (13)
Did quarantining due to COVID-19 symptoms, diagnosis, or exposure of self or household member impact the number of hours you worked as a PCA?	
No	75.5 (133)
Yes	23. 9(42)
Since the start of the pandemic, did you feel that any of the physical home environments you worked in were hazardous (hoarding, infested with pests, very unsanitary)?	
No	65.3 (115)
Yes	9.7 (17)
Has your household had any financial difficulties because of the COVID-19 pandemic?	
No	38.6 (68)
Yes	48.3 (85)
Not sure	9.7 (17)
Not including yourself, is anyone in your household at higher risk of complications from COVID-19 due to their age or health conditions?	
No	38.1 (67)
Yes	43.8 (77)
“My age or health put me at increased risk for severe complications from COVID-19.”	
Strongly agree	31.3 (55)
Somewhat agree	27.8 (49)
Neither agree nor disagree	23.3 (41)
Somewhat disagree	9.1 (16)
Strongly disagree	8.0 (14)
Benefits offered through job as PCA	
Paid leave (such as sick leave, personal days off, paid vacation days)	6.25 (11)
Extra pay or shift differential for working certain hours (e.g., holidays, weekends, or overnights)	7.4 (13)
Extra pay or bonus pay for working during the COVID-19 pandemic	12.5 (22)
Assistance with childcare (including facilitating arrangements or allowing you to bring your child to work)	1.7 (3)
Health insurance	5.1 (9)
Dental and/or vision insurance	4.0 (7)
Retirement contributions or pension plan	2.8 (5)

Categories of each variable may not add to 176 due to missing data.

**Table 6:** Self-reported health, quality of life, and mental health among PCAs.

	All (*N* = 176) %, (*n*)
Overall health	
Excellent	18.2 (32)
Very good	35.8 (63)
Good	34.7 (61)
Fair	9.7 (17)
Poor	0.6 (1)
Quality of life during height of pandemic	
Excellent	16.5 (29)
Very good	23.9 (42)
Good	33.5 (59)
Fair	16.5 (29)
Poor	5.7 (10)
Mental health during height of pandemic	
Excellent	12.5 (22)
Very good	17.6 (31)
Good	38.6 (68)
Fair	22.7 (40)
Poor	5.7 (10)

Categories of each variable may not add to 176 due to missing data.

### The commitment and call to care during the pandemic

PCAs were drawn to this work out of a desire to care for others, and while some left this field during the pandemic, many others remained largely out of a commitment to those they cared for. PCAs often formed deep bonds with their clients and felt personally responsible for ensuring they received safe, quality care during the pandemic. When asked to share anything else they would like us to know about their experiences as a PCA during the pandemic, one self-directed PCA survey participant added:

We choose to do this job because our hearts are in it not because we make money or get benefits. I could definitely get a higher paying job. I just do not have the heart to leave my client not knowing the care he will receive.

The connections and relationships that develop also motivate PCAs to continue this work, as shared by an interview participant (self-directed PCA):

There are no benefits for [PCAs]. The pay could be a lot better, but it pays in love.

An experienced agency-based PCA echoed this sentiment:

I love [my job]… the interaction with my clients and bein’ able to pretty much make their day and help them with whatever they need help with.

Some PCAs also felt particularly suited to caregiving, in that they both enjoy it and are good at it. A self-directed PCA who currently cares for her mom and uncle on the HCBS waiver, but has a long history of providing direct care in both nursing home and private home settings and has a goal of starting her own homecare agency, shared:

I love what I do. I love taking care of people. That’s what I do, is take care of people, even outside of my [family]… That’s just my passion, helping people. Always been that.

Related PCAs often shared that they will always care for their family members, no matter what. Many increased their caregiving hours either due to workforce shortages or concerns about increased risk of COVID-19 from outside workers. A related PCA discussed increasing the care she provided her son on the Brain Injury waiver due to being unable to find enough outside PCAs and despite her back pain:

It does not matter if [hurts] or not, I still have to do it. So if I have to work, you know a 16 hour shift… taking care of my son. It does not matter if he needs to be lifted, I have to lift him… I have to do it no matter what my physical condition is. There is nobody else to do it; it’s me. I have arthritis. I’ve had it… since my early 20s. Sometimes I hurt, but even if I’m hurt, I’m taking care of my son.

PCAs also noted their role in keeping their clients out of nursing homes, which was especially important during the pandemic when the virus spread rapidly in congregate settings, as demonstrated by these written responses to the open-ended survey questions “Why did you choose to continue working as a PCA during the COVID-19 Pandemic?”:

Because someone had to do it, no one would be available to take care of this population if everyone quit. I could get a job starting out at $15 but I think it’s important to be a caregiver. Because if we do not take care of them they’ll end up in nursing homes and die on us. As long as they stay in their home environments they live longer. (Agency-based PCA).

Because my client needs someone there and without us PCAs he would be in a nursing home being neglected and not cared for as needed. (Self-directed PCA).

PCAs sacrifice pay and benefits to remain in this line of work as several of the above quotes demonstrated. There were many other personal sacrifices made by PCAs who went above and beyond to provide quality care to their clients during the pandemic. It was not unusual for PCAs to report providing uncompensated hours of care, which while might be expected of the paid family caregivers, non-related PCAs also donated their time to client care needs. For example, an experienced self-directed PCA with multiple non-family-member clients shared:

The social workers and stuff wasn’t going into the homes or making home visits which made it difficult. They could not get the hours that they needed. Sometimes I’d stay over my time. I just clocked out when I needed [to], but if the job wasn’t done for the amount of hours they gave me, I went ahead and did it on my own free time because that’s just the way I am.

An agency-based PCA echoed

Sometimes I do stay a little longer than usual [and the approved hours]. I do not mind ‘cause I care for her so much…. it’ll only take me five or 10 min or something, and I’ll do it for her.

Some PCAs also put in additional paid hours to help cover shifts in the face of growing workforce shortages or when their colleagues were in quarantine. An agency-based provider for the Intellectual and Developmental Disability waiver shared:

We already are incredibly understaffed that’s just a national fact in our field, but this pandemic made it even more impossible…. and we got to points where we were having people work all night and all day trying to cover people who are incredibly ill and at their most vulnerable place because we have got no one.

However, for those in the self-directed program, they did not get paid overtime for these extra hours. Essentially, overtime rates are not approved in self-directed consumer budgets, and so to make overtime wages work on paper and by law, PCAs agree to having their base wages reduced, as shared by one PCA, “We actually cut my pay so that would fit within time and a half,” in which they end up working more hours for the same amount of total take-home pay. Several PCAs were frustrated by this but willing to put in these extra hours due to their commitment to care.

PCAs also provided unpaid supports that they felt were essential for their clients’ physical and mental health but were not approved services for billing. Pandemic specific examples included checking in and providing companionship by phone during quarantines, shopping for and dropping off supplies for clients in quarantine when there was no way to clock-in for this service, or supporting clients while they were hospitalized. A self-directed PCA survey participant wrote:

Yes, many of our tasks cannot be done virtually but out of care and concern for these clients we have built long-term relationships with, we did everything we could even when we could not be together in the same space.

A home care agency director lauded the various contributions she saw her staff make:

The pandemic helped us, once again, understand that our Direct Support Professionals truly are one of a kind when it comes to caring for our clients. Their concern for their clients outweighed personal time or financial costs. Caregivers would deliver casseroles and leave them on the doorstep or offer to do laundry if it was left on the doorstep or pick up groceries for clients.

Most PCAs took COVID-19 safety practices very seriously but also noted this made their jobs more difficult and stressful. Many described going to great lengths to keep their clients safe from COVID-19. For example, a self-directed PCA described changing her clothes between households:

I was precautious. Sometimes a little overly precautious…. Even in the cold weather, outside my front door before I go in my door… I would strip my clothes, my shoes, my panties, my underwear, everything off. Take that alcohol, rub it all over my body and put it in a bag. Then in my little suitcase that I carried with me, I’d re-change my clothes and my shoes.

Several PCAs described cleaning relentlessly, sometimes against the odds in homes with pests or hoarding issues. Some PCAs made personal sacrifices to keep their clients safe. For example, a related PCA shared her decision to continue her education remotely:

We all love people, but we just have to be cautious anymore. It’s sad to have to be that way, but it’s about trying to stay healthy…. I’m doing school online and stuff. I usually like to be on campus, but, since the pandemic, I try not to enroll in classes that’s on campus because there’s so many people. It’s just so risky…. I wanna be protected for [my clients]. I do not wanna bring it to them. If I’m going to school, and then I get it from school, and then have them at risk.

Another PCA detailed limiting her music therapy business, even though this was a higher paying job, to keep her clients safe:

I used to see a lot of people for a short amount of time, a lot of 30-min sessions. I feel like that’s not a safe choice because of the vulnerable people I see. I feel like for everyone involved, the safer thing is to do more caregiving at a lower rate of pay [than music therapy] to minimize exposure…. cause if I brought COVID to [my main client], he would probably die, and I would carry that with me the rest of my life.

Others described limiting their social bubbles to only their immediate household members and clients, at the expense of no longer seeing other close friends and family in person, as described by an agency-based PCA:

I kind of stayed away from relatives and different things because I knew I was working with older people. I would call them and talk to them, but I did not go and see them, or if I did see them, it was like go see them outside in the air, you know what I mean?

Another PCA, self-directed, described not being available to help care for her adult daughter with COVID because the PCA was unwilling to risk that exposure for the sake of her clients. Finally, many PCAs incurred out-of-pocket costs to provide safe care by purchasing their own masks and other personal protective equipment. A self-directed related PCA shared:

I pay for my own supplies. I even pay for my own sanitizer and my own [gowns], I do not depend on [my clients] to get anything…. I could not even tell you exactly how much I’ve been spending but… it adds up.

The survey data revealed that 33% of PCAs incurred out-of-pocket expenses to make the homecare services they provide safer.

### An undervalued and invisible essential workforce

PCAs remained in this field because they knew how important their work was for the health and wellbeing of their clients. Their value was widely recognized by their employers and clients, who often lauded PCAs as invaluable and the backbone of the HCBS system. Furthermore, PCAs surveyed overwhelmingly felt they were respected by clients as part of their home care team (72 and 16% strongly agreed or somewhat agreed with this statement). In contrast, however, interview participants felt this work often went unrecognized by the general public and undervalued by policy makers. An exceptionally committed PCA, who donated both time and financial resources to her clients, shared her frustration about being invisible:

When they were giving all these blessings and compliments to the nurses that are at risk in the hospital, they seemed to forget about us little people and the elderly there in the homes that are doing just as good as job as they were. We wasn’t even mentioned. We are a healthcare [worker, but] we were not recognized for nothing…. Even a client said we oughtta call up there, and we oughtta tell ‘em how good of a job you are doing…. I guess that was good enough ‘cause I knew they appreciated me, but it did kinda hurt my feelings.

A client on the Physical Disability waiver who is also a caregiver to her spouse with a brain injury was also frustrated by this oversight:

My recommendation is that the caregivers that came into these homes knowing that this pandemic was still out there, they risked their lives … for the people that they worked for…. I do not think they got recognition in that… ‘cause without them, we could not have stayed in our homes. We could not have been cared for as we were…. Give ‘em an award. Give ‘em a medal. Give ‘em a letter. Each state senator should send a letter to these people and caregivers and say, “Thank you for your duty. Thank you for caring for another person.”

This invisibility had real-life consequences for PCAs and their clients. It was not always clear if PCAs were essential workers, as shared by a client on the Physical Disability waiver:

When it first started, nobody was sure. I wasn’t sure. PCAs were not sure if they could go out because we were in lockdown.

Although state officials and providers confirmed that PCAs were essential workers, the lack of awareness of this workforce impacted access to key pandemic resources. As noted above, many PCAs purchased their own personal protective equipment. This reflects the fact that their employers had inconsistent access to these critical supplies or funding, as shared by a provider for the Intellectual and Developmental Disability waiver:

We felt very much like the forgotten stepchild, that the medical providers we are getting all of the support and [personal protective equipment] and we even had providers that… were scrutinized for having that [personal protective equipment] and told that we need to save those for hospitals. But we were expected to work directly with these clients, because they were not being hospitalized when they were diagnosed with COVID so our staff who are making close to minimum wage, a little bit above, are expected to work directly with COVID positive clients and we are also scrutinized for providing PPE for them so that was very overwhelming.

A home care agency provider indicated in an open-ended survey response:

It felt that as an agency that provides non-medical services… we were left out. Oftentimes we would find out after the fact about programs that would hand out donated gloves and other [personal protective equipment] items. Additionally, convincing the Health Dept. that our staff needed vaccinations was a problem.

This quote also illustrates how PCAs were not consistently recognized as a priority group for initial COVID-19 vaccination, as will be further detailed in a future paper.

The ultimate devaluing of this workforce is in their low wages and lack of benefits, as well documented in the literature and reinforced by our findings. Only 6 and 5% of PCAs surveyed in our study reported access to paid leave or health insurance, respectively, through their job. Most PCAs were insured through either public program (e.g., Medicaid, Medicare, or the VA) (29%) or a family members coverage (19%), but nearly 20% were uninsured. When only looking at PCAs ages 64 and under, who do not qualify for Medicare (the universal health insurance program for older adults in the United States), the uninsurance rate was 24%. For context, this is about twice the average rate of uninsurance for working aged adults in the United States (12%) (Cohen and Cha, 2023).

The invisibility of this workforce also impacted access to COVID-19 emergency funding to cover hazard pay, sick leave, or overtime for this workforce. The self-directed workforce had no access to hazard or sick pay, as clients were their employers and did not have feasible mechanism for accessing COVID-19 emergency funds for this purpose, as further detailed in Wendel et al. (2023). A self-directed PCA provided written comment on how this impacted her:

Having contracted COVID-19 [at] my [PCA] job, I had to self-isolate for 10 days, during which I could not work either of my jobs while I recovered. No income… my (unemployment) application was rejected… There was NO HELP from anyone. It was very frustrating AND demeaning. Not only were we at higher risk for exposure, when we got sick, we had no sick pay or unemployment benefits and shouldered the financial consequences on our own. (Emphasis in original).

Approximately 39% of participants surveyed reported being exposed to COVID-19 through their PCA job, and nearly 24% reported missing work hours due to needing to quarantine their self. It was also difficult for agency-based providers to access these funds. A small homecare agency detailed that her request for bonus pay, from county funds earmarked for the pandemic, was denied which she felt was because the county commissioners did not understand the nature of PCA work and the risks they took on:

I asked for bonuses… and the commissioners refused to give it to my three [PCAs]… We lost a very, very, very great [PCA] over it… and those [PCA] were still going into those homes… while risking themselves because some of the clients still had different people visiting them…. It was only going to be $500 or $1,000 for the [PCAs’] bonus and they refused to give it to them…. That was very difficult. I almost walked because of that, but I just could not do that to my staff, and boy, I hate to even think about it because it really, really, really upsets me.

The difficulty obtaining COVID-19 emergency funding for home-based services resulted in uneven access to COVID-19 benefits among this essential workforce.

A notable exception was the recruitment and retention bonuses utilizing American Rescue Plan Act funds, described above, which allotted between $1,500 and $2,000 per qualified PCA (Heydon, 2023). These funds were distributed relatively late in the pandemic and during our data collection period, during the fall of 2022, but were addressed in later interviews and surveys. PCAs and providers were grateful for these bonuses but also shared concerns. The state struggled to identify PCAs who were eligible for these bonuses, highlighting their invisibility in state data and communications systems, which resulted in a prolonged and convoluted process for distributing the bonuses. Clients and caregivers expressed frustration that former PCAs did not receive these funds, even though they provided essential care during the frightening early days of the pandemic. They also noted that the criteria for recruitment bonuses were not clear, and therefore, they did not advertise the bonus when recruiting new workers, as shared by a paid family caregiver who was also trying to hire external PCAs:

They did not explain a lot of the details. I could tell you for certain there was a lot of confusion on a lot of the Facebook parent groups that I’m on. People were saying, “I was told this.” Somebody else was like, “No. That’s not right, I just talked to our [Targeted Case Manager], and they said this.” Somebody else said, “That’s not right either.”

A self-directed PCA who worked throughout the pandemic stressed that while every dollar helps, the bonus was not nearly enough to offset costs she incurred:

The fact that all those (personal protective equipment) supplies are out of pocket is a hindrance… There’s no PTO (paid time off). If I get exposed to COVID or my roommate did, that was two weeks I lost of income, [that happened] at least three times, so six weeks [unpaid].

Finally, some agency providers shared they were not aware of the bonus or found out too late, and, therefore, never received these funds for their PCAs.

PCAs as experts on their clients’ care needs were also not well recognized or supported. Care coordinators, case managers, and eligibility assessors all moved to virtual contact only, with PCAs then often being the only remaining professionals who had direct contact with HCBS clients. PCAs were the eyes and ears of the HCBS system, yet the system was not set up to accept their feedback. Many PCAs did not understand how the HCBS system operates, including how their wages are funded, how care plans are set up, or where to take their concerns. Yet, the few PCAs who were system-savvy expressed frustration that their concerns were ignored, as demonstrated by an experienced self-directed PCA:

I’m not that knowledgeable about what’s on their record or in their file. All I know is what their needs are…. I put my job on the line out there to do things. I had to do them because I wasn’t getting no help for these people. These people (care coordinators) wasn’t listening to the clients’ needs… When we were out here being their eye and the voice, they would not listen to us.

Another PCA reached out to state officials and legislators about her system-level concerns, only to be dismissed because her name was not in the provider registry, indicating a fundamental lack of knowledge of the PCA role and their invisibility in the system. She also noted that public stakeholder feedback opportunities were designed for providers or family caregivers and not PCAs.

### Emotional toll of direct care work during COVID-19

The daily work of PCAs is challenging on a “normal” day, but during the pandemic, these challenges were magnified. PCAs expressed intensified feelings of fear for the health and safety of their clients, and these heightened anxieties exacted an emotional toll on PCAs during the pandemic. These challenges are built on top of the low wages and lack of benefits described above. This emotional toll, the unrelenting nature of their work, limited resources, lack of respite care, and lack of institutional support led some PCAs to experience burnout.

A majority of PCAs (61.3 percent) described their mental health during the pandemic as “fair” to “good,” and 57.4 percent described their quality of life as “good” to “very good” during the pandemic. Despite the survey data indicating that mental health of most PCAs fared well during the pandemic, interviews uncovered specific areas of anxiety and concern. The work of PCAs is physically, mentally, and emotionally challenging as described by an agency-based PCA:

It’s hard work… I do not think people understand how hard it can be because we go in there every day and take care of people that have different personalities. They have different medical needs, and we have to make sure that they have everything that they need, and that we have everything we need…. I’ve been pinched. I’ve been hit. I’ve had my hair pulled. I’ve been kicked.

Given that PCAs provide services within the client’s homes, they also face the associated risks of working within the client’s social network and home environment. PCAs described carrying out care tasks in homes that were dangerously unclean. An agency-based PCA shared:

There are occasional houses that we go into that do hoard and it’s hard to keep everything clean and sanitized. And we have had a couple that I’ve had roaches and we have had an increase of people with bedbugs lately.

Unrelated PCAs have an intrinsic boundary to protect their personal and professional lives by virtue of their scheduled hours. Related PCAs, especially those that live with their care recipients, do not have this same intrinsic boundary. It is often difficult for related PCAs to find times when they are not “on the clock.” A related PCA shared the unrelenting nature of her care work,

I have to manage everything. I have to tell the insurance company when they are not paying things correctly. I have to tell school districts when they are not following the law. I have to tell doctors when they are not doing what they need to do… I do not know if people realize that how much, when you are the caregiver, you have to run things. It’s exhausting.

The pandemic intensified these challenges primarily due to fewer PCAs to share the care work load with, increased safety measures, the social isolation of quarantining and social distancing, and the fear of the unknown of contracting and spreading the virus. Fear of catching or spreading COVID-19 to their vulnerable clients was a common sentiment and source of anxiety for PCAs. An agency-based PCA described her fear at the start of the pandemic and when returning to work after the lockdowns:

A lot of people were dying. The hospitals were filled. I was scared about getting it, and then, also, I was really also scared about giving [it] to someone else. Personally, for me, if I knew I had COVID and I gave it to someone else… and they died, I would, literally, probably never forgive myself. I would feel so bad… I was really nervous to go back to work in general.

To limit exposure to the virus while caring for clients, PCAs often adopted strict safety guidelines beyond what their employers required, as described above. However, their ability to control the spread of the virus was limited by the choices and behaviors of their coworkers, clients and their family members, and other social networks. A self-directed PCA described how her client’s other PCA had exposed not only the client but also infected other care team members with the virus:

I’m gonna say that [my client] had one staff that exposed 11 people… [by] not wearing [a] mask… I was pretty upset. I’m very protective about our people. That’s part of our job is to ensure their safety.

A PCA from the survey shared that the client’s home environment exposed her to COVID-19, which was the most challenging part of the pandemic for her:

Despite taking all precautions, contracting COVID-19 most certainly from my client/her senior apartment building, where management and residents did not take full precautions.

Not being able to rely on others to take pandemic-era safety seriously potentially held higher costs to PCA wellbeing and financial livelihood than the general population, due to the lack of benefits described above; as one PCA shared, “Other people’s decisions greatly affect me.”

When clients ultimately contracted and lost their battle with the COVID-19 virus, PCAs had to manage their own grief while continuing to protect themselves and care for other clients. A self-directed PCA shared,

We lost a customer… It was like I shoved it all down, and I came to work. I was like, ‘I’m sick from shoving my emotions on the side.’ … It has gotten better, but it’s a lot some days.

PCAs not only had to handle their own anxiety about keeping themselves and others safe during the pandemic but also had to help manage the emotions of their clients. HCBS clients who thrive on strict daily routines or social engagement were suddenly faced with drastic changes to their everyday life. Social isolation was a particularly difficult challenge for many clients, and workers were often the only interaction clients had with the “outside” world, as shared by a survey respondent:

Telling the individuals they could not see their family, while driving home to my family every night. It changed me. Being the bad guy to people who did not understand. I saw behaviors in individuals that were extremely out of character because they missed their families.

The pressure of being a clients’ only source of support and socialization led PCAs to experience a sense of role overload which negatively influenced their job performance and led to burnout. In addition to long, unrelenting work hours, PCAs felt guilty for setting boundaries between their work because their clients were often isolated from their social networks. A PCA shared:

I was taking care of people three hours during the day and four hours during the early afternoon, four or five hours in the evening up until midnight. Then I had a client that I did night shift all night long. [I was] tired but I did [it]…I’m in between a rock and a hard spot. This is my passion. I also feel like if I’m not there, [then] nobody else will be there…there’s just me… I had some of [my clients say], “Please do not go. Could you spend a little bit more time with me? I’m tired of being’ here alone.”

In addition, many related PCAs reduced the help they received from external workers, as explained by a PCA to her daughter on the Brain Injury waiver:

I did not want people in my house. I mean, nobody understood COVID yet and how it was transmitted, and I said, stop. I do not want people coming in my-people are dying in the hospital, and you know… Everybody was afraid, including me. I mean, I’m my daughter’s caregiver, I do not want to die because I got COVID. I said, No [PCAs] right now until this is figured out.

As a result, related PCAs received less respite in their caregiving role.

The work-related stress and emotional experiences of PCAs during the pandemic was neither static nor isomorphic across the workforce. Overall, PCAs surveyed did not report high stress levels, with only approximately 23% indicating their jobs were always or often stressful. Furthermore, over half of survey respondents indicated that their job-related stress was the same as before the pandemic. This contrasts with interview participants, who more typically described job-related stress as increasing during the pandemic. Several PCAs interviewed described experiencing more anxiety and depression early in the pandemic, but after vaccines became available and social distancing lessened, work-related stress lessened. PCAs described their work during the pandemic as meaningful, providing them a sense of purpose in knowing that they were providing an essential service to others during a global crisis. One PCA shared:

It gets me going every day, for one thing. I know people depend on me. Helping them makes you feel good—and their friendship. It’s just good for me.

The diverse experiences of PCAs during the pandemic may reflect the support systems and self-care strategies that PCAs utilized during the pandemic. Coworkers were found to be sources of support primarily because they understood exactly what each other were going through during the pandemic. A PCA wrote, “My coworkers were always a shoulder to cry on when days were unbearable.” Faith was also an important coping strategy shared by PCAs. Professional mental health services also provided much needed support for PCAs. An insured agency-based PCA stated:

I did get some bad anxiety and depression [during the pandemic], so I was able to go to [therapy]. That helped a lot. Getting therapy helped a lot.

Some agency-based employers provided important support by providing COVID guidance and emotional support. A PCA shared that her financial management service provider distributed small tokens of appreciation:

At one point, during lockdown, they sent us little packages with paperclips, a journal with little positive thoughts in it. Just little simple things. A card that said you were appreciated. [My company] is very good about letting you know we appreciate you. We’re blessed with a very special CEO. They do so much.

However, both agency-based and self-directed employers varied widely in the support provided to PCAs. Approximately 32% of PCAs surveyed strongly agreed or somewhat agreed that their employer provided good support and assistance during the pandemic, 6% disagreed, and 12.5% neither agreed nor disagreed. When we asked PCAs the open-ended question, “What was the most important thing others have done to support you in your role as a Direct Support Worker during the pandemic?” few respondents pointed to tangible supports, but several mentioned emotional support or feeling appreciated by their clients and their family. However, among those who provided a response, over a quarter indicated there was no support, with written responses such as “Umm… nothing” and “What support?”

### Impact on job satisfaction and worker quality

Participants overwhelmingly found their roles as PCAs to be meaningful and fulfilling, as shared above. Participants discussed how they feel called to do this work and can support clients when others cannot. One caregiver shared their experiences with PCAs:

The individuals that are out there are just wonderful. They really are! They truly are doing the work that they are doing because they care about these individuals. So, [the providers] really, really have been able to assemble a really wonderful team of people. It’s just there aren’t enough of them.

Clients and their families rely on PCAs to be passionate about their jobs and emotionally invested in the support they provide to clients. One PCA shared,

This is my passion, and I like to care for the people. I’m happy that I was there for them when nobody else would be.

Yet, finding PCA work fulfilling is not always enough by itself to be satisfied with their jobs.

When asked about how satisfied they are with their jobs, responses were mixed across surveys and interviews. Approximately 89% of survey participants reported that they were extremely satisfied or somewhat satisfied with their jobs. During interviews, when asked whether they were satisfied with their job, PCAs typically provided a nuanced answer, as succinctly stated by one PCA, “With the job, but not the pay.” Another PCA shared conflicting feelings about their job:

I was not satisfied… I was like this is not good. I’m caring for people and I do not like it… There’s a forum on Reddit, people would be like, ‘I’m burnt out. What do I do?’ Or they are like, “I hate my job.” It’s like I’m not the only one…. I think the reason why I do not like this job is because, one, the pay is way too low. That is unreasonable. I’m not trying to compare jobs or anything like that, but if my friend who works at Target gets paid $3 an hour more—she gets $13.50, $14 an hour, while I get paid $10 an hour or $9 an hour at the beginning, I was like, I’m literally cleaning up stuff. I’m peri-care. I’m doing these medication regimens. I was like, ‘This is crazy.’

This PCA eventually left the field and noted her new job pays much more and is far less stressful. Participants expressed widespread dissatisfaction with the low pay and poor benefits associated with the job. Approximately 46% of PCAs surveyed reported experiencing financial difficulties due to the COVID-19 pandemic, and as shared above, access to paid leave was minimal. One participant shared their thoughts about benefits, particularly the importance of paid time off,

I think we should get paid more or get some type of benefits. They do not offer no insurance, like I said, no sick days, no vacation. I have not had a vacation, which I take, so I have to take a vacation with no pay. Because you need the time off and I have not had time off. I was in the hospital, maybe about a month ago…when I came out of the hospital I just took the week off …. I’m like, ‘I gotta recuperate, get myself together’. You need time out, everybody needs a vacation.

There was an important counter trend to the dedicated PCAs who felt called by caregiving and committed to the safety of their clients during COVID-19. To begin with, many PCAs quit in the face of low wages, leading to workforce shortages that are well documented in the literature. Seven percent of survey respondents said they were “very likely,” and 16.4% said they were “somewhat likely” to leave the PCA workforce in the next year. A client-employer described losing a PCA due to lack of benefits,

She [former PCA] had a really good personality. She was jolly to be around. She did a fabulous job. She would come if I needed something after she had gotten home. She would come back over, just a very good person…. She left (during the pandemic) for full-time employment with benefits.

Another client-employer shared:

I honestly did hire one outside person. Oh, I loved her. She was about the best caregiver I’d had in my entire life. Then she recently quit [because of] burnout.

The well-qualified PCAs who left the field were difficult to replace, with clients and caregivers instead finding they were left with a pool of poor workers. Some respondents spoke of PCAs who simply did not do the work they were hired for, while others pointed to more serious concerns, as indicated by a related PCA in managing outside PCAs on her son’s care team:

We had some pretty interesting folks come through. I mean, we were at the point where we had to give the job to whoever applied, or we were doing it ourselves. I mean, we are talking—I’ve had caregivers leave their drug paraphernalia in my house. I’ve had people steal from there. I mean, all kinds of stuff.

A self-directed client described having their property and medications stolen:

I’ve had the pleasure of having—I’ve had my car stolen, I’ve had money stolen, I’ve had my meds stolen…. I’ve had one that tried to blackmail me into getting my meds… and she was a CNA (Certified Nursing Assistant), so I thought it would be a heck of a good fit. I was totally wrong on that…. With my granddaughter, I’m not getting [all] the hours or what I need done, done, but at least I do not have to worry about her stealing anything…. If you had better pay, you would get better quality of people, I hope.

As noted by one self-directed client, “you get what you pay for,” low wages limited their choice of workers and some felt they were stuck with workers rejected from other jobs. Furthermore, some expressed concern that the private home environment attracted PCAs who preyed on this vulnerability.

The shortage of quality workers puts clients and their families in the difficult position of needing to weigh their need for PCAs to provide care and support to remain in the community against the risk of allowing questionable PCAs into their home. Ultimately, clients and caregivers fired workers who engage in bad behavior, such as stealing or providing subpar care, although sometimes with delay or hesitation due to the workforce shortages and concerns about being able to replace these workers. One financial management service provider explained:

That’s a problem. People keep—have kept workers that they really did not want to keep because they could not find anybody else. That’s still a problem today.

Finally, many family caregivers felt they did not really have a choice other than to continue serving as a PCA for their loved ones. A few shared that they would prefer outside help but cannot find anyone in the face of the workforce shortages, as shared by one mother:

I did not choose [this occupation]. My daughter receives PCA hours, and we have been unable to find a worker for her…. We would much prefer that over me getting paid.

These family caregivers often noted sacrifices to their career, including earning potential, or that caregiving duties have become increasingly difficult for them in light of their age or own health conditions. Another family caregiver for an adult son with a brain injury noted she would prefer to focus on the emotional bonds of motherhood while delegating the more intensive caregiving duties to outside PCAs, sharing:

I only fill in, yeah. I do not have any set hours for me…. I wanna be mom. I do not wanna be [the] caregiver… I do it when I have to.

## Discussion

Study results demonstrate the commitment of PCAs to care for older adults and individuals with disabilities during the COVID-19 pandemic, despite low wages, inadequate benefits, and limited support. PCAs often went above and beyond to provide quality care and keep their clients safe during this time but were not well recognized, rewarded, or supported as essential workers.

Our findings on job stress and satisfaction were nuanced. While PCAs in the interview sample described the increased stress and strain of working during the pandemic, surveyed PCAs indicated that, overall, work-related stress had stayed about the same in comparison with before the pandemic. This may be due to the non-monetary, emotional rewards of this work. PCAs described that while the workload can be intense and challenging, their work also provides them with a sense of purpose, importance, and motivation. In other words, the benefits balance out the emotional costs. This may be the very factor that privatized healthcare systems rely on to justify paying PCAs less, knowing that PCAs feel a strong sense of dedication and commitment to their clients despite the emotional, financial, and physical toll of this work. Similar arguments have been made about the exploitation of ‘caring’ among teachers (McKittrick-Sweitzer, 2023), and home care companies market the compassion and warmth of their employees to clients (Franzosa and Tsui, 2020). Similarly, Folbre (2001) highlights how care workers become “prisoners of love,” in that they are often unwilling to quit or strike in the face of poor working conditions because extended absences would threaten the welfare of those they serve, which reinforces their low wages. However, growing PCA workforce shortages demonstrate that relying on those who are dedicated to care work despite low wages is inadequate to meet the growing demand for home care.

Burnout among PCAs is one reason for the caregiving crisis facing the United States (Green, 2022). Particularly for PCAs, the combination of low wages, lack of employer benefits (such as paid time off, health insurance, and hazard pay), the emotional and physical demands of their work, and lack of recognition are primary markers for occupational burnout. Maslach (Maslach and Jackson, 1981) identified three primary characteristics of occupation burnout: emotional exhaustion, depersonalization, and lacking a sense of personal accomplishment or having autonomy and voice within one’s occupation. PCAs in our study repeatedly described instances of feeling overwhelmed and emotionally exhausted during the pandemic, and despite seeing their work as worthwhile and rewarding, the low pay, benefits, and recognition prompted some to seriously contemplate leaving their position.

The respondents in our study engaged in various self-care activities and found support through their social networks of clients, coworkers, friends, and family; however, these internal practices only protect workers against occupational burnout to a point. External or institutional factors such as flexible schedules, fair wage and benefit structures, growth opportunities, having a voice in decision-making within the organization, and availability of peer, supervisor, and training supports help to prevent and mitigate occupational burnout for workers (Rehder et al., 2021). Rehder et al. (2021) argue that PCAs who engage in quality self-care practices and possess resilient psychological attitudes toward their work can still experience burnout if they work in continuously toxic, unsupportive work environments. Similarly, PCAs, who work in supportive environments, who lack self-care strategies, and hold a negative stance toward their work, can still experience occupation burnout. While a majority of PCAs felt respected by individual clients as part of the care team, the lack of larger structural supports and societal acknowledgment of the work that they do contributed to ambivalent feelings about their work. Protecting PCAs against burnout, therefore, requires both individual and institutional support factors to prevent and mitigate occupational burnout (Gray-Stanley and Muramatsu, 2011; Boerner et al., 2017; Rehder et al., 2021).

In supporting individuals with complex care needs, PCAs have professional-level responsibilities but without the pay, recognition, or influence of a professional healthcare worker. The United States missed a key opportunity to invest in this workforce when over $400 billion in funding for in-home care was removed from the Infrastructure and Jobs Act (Higgins, 2021). The inclusion of care workers as human infrastructure in the proposed bill was a novel approach, reflecting a recognition of not only the value of caring for children, individuals with disability, and older adults, but also the role of paid caregivers in allowing family caregivers, especially women, remain in the workforce. However, conservative lawmakers pushed back against the notion that care workers were vital to the nation’s infrastructure and economy, and this funding was removed in the bipartisan compromise to pass the bill (Li and Laughlin, 2023). The failure to recognize care work as vital to the nation’s infrastructure illustrates that, as long noted by feminists theorists on caregiving (e.g., Tronto, 2013; Fraser, 2016; Folbre, 2024), policy makers continue to neglect the contributions of caregiving to our economy. This further demonstrates the importance of taking this growing workforce out of the shadows and educating policymakers on their essential role, an important step in enhancing the recognition of this workforce (Lyons and O’Malley Watts, 2024). Most everyone will eventually need LTSS as they age (Johnson and Dey, 2022), but unless critical investments are made in this workforce, a growing number of Americans will find that this care is not available for themselves or their loved ones when they need it.

Professionalizing the PCA workforce also entails recognizing the expertise of PCAs as knowledgeable members of the care team. PCAs have frequent, hands-on contact with their clients and, during the pandemic, were often the only healthcare professionals with this face-to-face contact, yet we found they struggled to make their voices heard when advocating for client care needs. Many efforts are underway to professionalize this work force through skills development and training [Centers for Medicare and Medicaid Services (CMS), 2023; Lyons and O’Malley Watts, 2024], but there is also a need for program development and evaluation on practices that can support the meaningful contribution of PCAs in care planning and system advocacy (Stone and Bryant, 2019). In addition to job skills development, PCAs would benefit from education on the HCBS delivery system, the role of the care plan, and how to communicate effectively with other healthcare providers. Formalized systems also need to be developed to permit PCAs to actively engage in care plan development.

The strengths and limitations of our study point to directions for future research. A key strength of this study was collecting data from stakeholders differentially situated in the HCBS system by including PCAs, family caregivers, clients, and providers. The data we collected from PCAs, especially the interview data, are likely biased toward high-quality workers, who were motivated by their passion for this work to also participate in this study. However, in also collecting the perspective of clients and family caregivers, we gained insight into the problem of poor quality PCAs. Our findings point to a polarization of the workforce, with a sharp divide between the high-quality, self-sacrificing PCAs and those who neglect or exploit their clients, and illustrate how the workforce shortage forced many clients and caregivers into the difficult choice of no care or poor care. The literature has well-documented the dedication of PCAs, but more research is needed on the “bad apples” in this field and the difficult decisions self-directed clients face as employers, to identify strategies needed to prevent and mitigate abuse, exploitation, and neglect.

Our interview and survey data were collected over a 26-month time span within a constantly evolving pandemic context, which had both advantages and disadvantages. This allowed us to see how shifts in COVID-19 infection rates, availability of personal protective equipment or vaccines, and the policy response impacted the everyday work of PCAs. However, it also sometimes made it difficult to compare and contrast experiences when data were collected at different time points in a rapidly changing situation. Ultimately, however, the ever-changing nature of the pandemic was the real context within which PCAs were performing their jobs. Another limitation is that while clients, caregivers, and providers spoke to high turnover rates, very few former PCAs who quit their jobs during the pandemic participated in our study, despite efforts to recruit them. Therefore, it is possible we did not capture the full experience of PCAs, especially those who were most dissatisfied with their work during the pandemic. There is a need for longitudinal research on this workforce to better understand how the changing social and policy environment shapes their job experiences and factors that influence decisions to leave this field.

## Data Availability

The raw data supporting the conclusions of this article will be made available by the authors, without undue reservation.
